# Implications of human evolution and admixture for mitochondrial replacement therapy

**DOI:** 10.1186/s12864-017-3539-3

**Published:** 2017-02-08

**Authors:** Lavanya Rishishwar, I. King Jordan

**Affiliations:** 1 School of Biology, Georgia Institute of Technology, Atlanta, GA USA; 2 PanAmerican Bioinformatics Institute, Cali, Colombia; 3 Applied Bioinformatics Laboratory, Atlanta, GA USA

**Keywords:** mtDNA, Population genomics, Three-person baby

## Abstract

**Background:**

Mitochondrial replacement (MR) therapy is a new assisted reproductive technology that allows women with mitochondrial disorders to give birth to healthy children by combining their nuclei with mitochondria from unaffected egg donors. Evolutionary biologists have raised concerns about the safety of MR therapy based on the extent to which nuclear and mitochondrial genomes are observed to co-evolve within natural populations, i.e. the nuclear-mitochondrial mismatch hypothesis. In support of this hypothesis, a number of previous studies on model organisms have provided evidence for incompatibility between nuclear and mitochondrial genomes from divergent populations of the same species.

**Results:**

We tested the nuclear-mitochondrial mismatch hypothesis for humans by observing the extent of naturally occurring nuclear-mitochondrial mismatch seen for 2,504 individuals across 26 populations, from 5 continental populations groups, characterized as part of the 1000 Genomes Project (1KGP). We also performed a replication analysis on mitochondrial DNA (mtDNA) haplotypes for 1,043 individuals from 58 populations, characterized as part of the Human Genome Diversity Project (HGDP). Nuclear DNA (nDNA) and mtDNA sequences from the 1KGP were directly compared within and between populations, and the population distributions of mtDNA haplotypes derived from both sequence (1KGP) and genotype (HGDP) data were evaluated. Levels of nDNA and mtDNA pairwise sequence divergence are highly correlated, consistent with their co-evolution among human populations. However, there are numerous cases of co-occurrence of nuclear and mitochondrial genomes from divergent populations within individual humans. Furthermore, pairs of individuals with closely related nuclear genomes can have highly divergent mtDNA haplotypes. Supposedly mismatched nuclear-mitochondrial genome combinations are found not only within individuals from populations known to be admixed, where they may be expected, but also from populations with low overall levels of observed admixture.

**Conclusions:**

These results show that mitochondrial and nuclear genomes from divergent human populations can co-exist within healthy individuals, indicating that mismatched nDNA-mtDNA combinations are not deleterious or subject to purifying selection. Accordingly, human nuclear-mitochondrial mismatches are not likely to jeopardize the safety of MR therapy.

**Electronic supplementary material:**

The online version of this article (doi:10.1186/s12864-017-3539-3) contains supplementary material, which is available to authorized users.

## Background

Mutations to mitochondrial DNA (mtDNA) have been associated with a wide range of human diseases [1, 2]. Since mitochondria are maternally inherited, mitochondrial genetic disorders will be passed from mothers to their children. Effective treatments for mitochondrial disease are rare, and patients are often faced with limited therapeutic options. Furthermore, the ability to accurately assess the risk of inheriting a mitochondrial genetic disorder can be complicated by the co-occurrence of wild-type and mutated mtDNA (i.e., heteroplasmy) in a single female [3]. Mitochondrial replacement (MR) therapy is a promising new assisted reproductive technology that could allow women with mitochondrial disorders to give birth to healthy children to whom they are closely genetically related. MR therapy works by combining nuclear DNA (nDNA) from a mother who has a mitochondrial disorder together with healthy mitochondria from an egg donor. For MR-assisted in vitro fertilization (IVF), the nuclear genome is removed from a fertilized oocyte with diseased mitochondria and injected into an enucleated donor egg that contains healthy mitochondria. This process results in so-called ‘three-person babies’ since children born from MR therapy will have genetic contributions from two mothers and one father.

Studies in mammalian systems over the last decade have underscored both the surmountable technical challenges, and the considerable promise, associated with MR therapy. The nuclear transplantation procedure that underlies MR therapy was first shown to be possible in mice [4]. The progeny of nuclear transplantations from mouse oocytes with mitochondrial disease into healthy oocytes were found to be viable and disease-free. Later in primates, MR therapy was used to produce four Macaque offspring [5] that showed healthy development to 3 years of age [6]. Nuclear transfer in this case occurred prior to fertilization, a technique that has not proven to be equally effective in humans. The MR procedure was first developed in humans using abnormally fertilized zygotes [7]. Human MR experiments rely on pronuclear transfer, whereby the nuclear genome is removed from a newly formed human embryo shortly after fertilization. This approach showed promise with respect to both the small amount of diseased mitochondria that are carried over to the healthy donor egg and in terms of normal in vitro development through the blastocyst stage. More recently, the same group demonstrated even greater efficacy of the pronuclear transfer technique for MR therapy with normally fertilized embryos by transferring the pronuclei compartments containing maternal and paternal haploid genomes almost immediately after they first appear [8].

These crucial experimental advances in MR therapy have occurred against a backdrop of substantial regulatory investigation related to the technique’s desirability, safety and potential efficacy [9]. Most of the effort and progress on this front has occurred in the United Kingdom (UK). The UK’s Human Fertilisation and Embryology Authority (HFEA) was initially charged with evaluating MR therapy, and they recommended further studies before the technique could be adopted as a clinical practice. Subsequently, several independent UK science agencies supported the bioethics of MR therapy, and the public was found to be largely in favor of its use. These findings ultimately led the UK government to draft a set of regulations for the technique, and parliament approved MR therapy as an assisted reproductive technology in February of 2015. Regulations were to be enacted by October of the same year, with clinics able to apply for a license by November. Initial attempts to conceive via MR-assisted IVF could have begun by the end of that same year. When this manuscript was written, there was no record of any human birth resulting from MR therapy in the UK. However, during the manuscript review process, news broke of a ‘three-person baby’ resulting from MR therapy born in Mexico [10]. The United States (US) doctors who led the procedure chose Mexico to avoid US regulations that do not yet permit MR therapy, leading to charges of unethical and irresponsible behavior.

Despite the considerable technical and regulatory progress that has been made on the issue, substantial concerns have been raised about the safety of MR therapy [9, 11]. These concerns rest largely on the notion of potential incompatibility (mismatches) between nuclear and mitochondrial genomes from different populations of the same species. We refer to this idea here as the ‘nuclear-mitochondrial mismatch hypothesis’. Incompatibility between nuclear and mitochondrial genomes from divergent populations would most likely be predicated upon interactions between proteins encoded by each [11]. While the human mitochondrial genome only encodes 37 protein coding genes, there are more than a thousand nuclear genes that encode proteins involved in mitochondrial function. These include 76 nuclear encoded proteins that directly bind mitochondrial counterparts. Sequence variations that change the binding affinities between nuclear and mitochondrial proteins could have deleterious effects, which would jeopardize healthy outcomes from MR therapy. This possibility has been supported by comparative sequence analyses showing the importance of compensatory sequence changes that serve to maintain physical interactions between nuclear and mitochondrial encoded proteins [12, 13].

A number of studies from model organisms have provided even more direct evidence for incompatibility between nuclear and mitochondrial genomes brought together from different populations of the same species. For example, mice with mismatched mitochondrial and nuclear genomes were able to survive to adulthood but showed stunted growth and reduced physical performance [14] as well as reduced learning and exploratory behavior [15]. The neurological effects observed in the latter study increased with age. Analogous studies in invertebrates have also turned up numerous deleterious effects of combining divergent nuclear and mitochondrial genomes. Such effects include changes in aging [16–19], survival [20] and fertility [21–24] along with impaired mitochondrial function [25, 26]. It should be noted that all of these studies entailed repeated genetic backcrosses whereby divergent mitochondria were introduced into highly inbred lines. As such, they represent extremes of genetic divergence between lineages and are not likely to accurately reflect human populations that routinely interbreed [27]. Nevertheless, these findings do point to a number of possible complications arising from nuclear-mitochondrial genome mismatch.

While the aforementioned studies have revealed instances of nuclear-mitochondrial incompatibility by studying the progression of chimeric individuals into adulthood, MR studies in humans have been conducted in vitro and only followed embryos through the blastula stage of development. This has led to calls for additional preclinical trials of MR therapy with a much longer time horizon [9]. However, it has occurred to us that long term experiments of this kind have already been conducted in nature via the process of human evolution. The evolutionary history of anatomically modern humans has been characterized by relatively long periods of isolation and genetic divergence interspersed with migrations and genetic admixture between previously isolated populations [28–30]. Admixture between genetically distinct human populations should have brought together divergent nuclear and mitochondrial genomes. Evidence that this is in fact the case could be taken to refute the nuclear-mitochondrial genome mismatch hypothesis for humans and to thereby support the feasibility of MR therapy.

In light of this realization, we systematically evaluated the extent of naturally occurring nuclear-mitochondrial genome swapping that has occurred among human populations. The goal of our survey was to get an idea of the extent of nuclear-mitochondrial divergence that can be tolerated within any single individual as well as a sense of how often swapping has occurred in human evolution. To do this, we evaluated the distribution of nuclear genomic diversity and mtDNA haplotypes among the 26 human populations, representing five major continental groups, which were characterized via whole genome sequencing as part of the 1000 Genomes Project (1KGP) [31]. We also performed a confirmatory analysis of mtDNA sequence variation among 58 populations from the Human Genome Diversity Project (HGDP), which characterized mitochondrial genomes to a lower level of resolution using SNP arrays [29]. We reasoned that since the donors for these human genome diversity projects are (apparently) healthy individuals, they should not bear incompatible nuclear-mitochondrial genome combinations. In addition, since these diverse populations have been shaped by millennia of natural selection, deleterious combinations of nuclear-mitochondrial genomes should have been eliminated long ago and would not be observed in extant populations. In this sense, our survey can be considered as a test of the nuclear-mitochondrial genome incompatibility hypothesis in humans.

## Methods

### Human population genomic data

Human genome sequence variants, characterized via whole genome sequencing of healthy donors, for nuclear DNA (nDNA) and mitochondrial DNA (mtDNA) were obtained from the 1KGP [31] data portal [32]. The data analyzed here correspond to the Phase 3 release of 1KGP [31], with variants available for 2,054 individuals from 26 populations representing five major continental population groups (Table 1). For the purposes of this study, we consider the ASW and ACB populations to be members of the Admixed American continental population group.

**Table 1:** 1000 Genomes Project (1KGP) populations analyzed in this study

	Short	Full description	*N*		Short	Full description	*N*
Africa (*n* = 504)	ESN	Esan in Nigeria	99	India (*n* = 489)	BEB	Bengali in Bangladesh	86
	GWD	Gambian in Western Division, The Gambia	113		GIH	Gujarati Indian in Houston, TX	103
	LWK	Luhya in Webuye, Kenya	99		ITU	Indian Telugu in the UK	102
	MSL	Mende in Sierra Leone	85		PJL	Punjabi in Lahore, Pakistan	96
	YRI	Yoruba in Ibadan, Nigeria	108		STU	Sri Lankan Tamil in the UK	102
East Asia (*n* = 504)	CDX	Chinese Dai in Xishuangbanna, China	93	America (*n* = 504)	ACB	African Caribbean in Barbados	96
	CHB	Han Chinese in Bejing, China	103		ASW	African Ancestry in Southwest US	61
	CHS	Southern Han Chinese, China	105		CLM	Colombian in Medellin, Colombia	94
	JPT	Japanese in Tokyo, Japan	104		MXL	Mexican Ancestry in Los Angeles, California	64
	KHV	Kinh in Ho Chi Minh City, Vietnam	99		PEL	Peruvian in Lima, Peru	85
Europe (*n* = 503)	CEU	Utah residents with NW European ancestry	99		PUR	Puerto Rican in Puerto Rico	104
	FIN	Finnish in Finland	99				
	GBR	British in England and Scotland	91				
	IBS	Iberian populations in Spain	107				
	TSI	Toscani in Italy	107				

The continental population groups, short three letter symbols, full population name and number of individuals from each population are shown. The continental population groups correspond to the convention used by the 1KGP with the exception of the ASW and ACB populations, which we consider as part of the admixed American population group

HGDP mtDNA sequence variants from healthy donors characterized via SNP arrays [29] were obtained from the HGDP-CEPH Genome Diversity Panel Database (version 3.0) [33]. The HGDP data analyzed here correspond to the Dataset 2 release from September 2007, with variants available for 1,043 individuals from 58 populations representing six major continental population groups (Additional file 1: Table S1).

### Nuclear and mitochondrial genetic divergence

Genetic divergence levels between pairs of individuals, for nDNA and mtDNA, were measured as allele sharing distances [34] as implemented in PLINK v1.90 [35]. Allele sharing distances are calculated as the number of different variants (*d*) normalized by the total number of sites (*2n*) under consideration. The resulting nDNA and mtDNA pairwise distance matrices were projected in two dimensional space using multi-dimensional scaling (MDS) [36] implemented in R [37, 38]. Allele sharing distances for nDNA and mtDNA were used to reconstruct a neighbor-joining phylogenetic trees [39] using the program MEGA [40]. nDNA versus mtDNA allele sharing distances were regressed, and the resulting scatterplot was visualized using a smoothed color density representation in R. The Spearman correlation coefficient was used to quantify the correlation between nDNA and mtDNA distances and the significance of the relationship. Genetic divergence levels for nDNA and mtDNA were ranked separately, and the ranks were compared in order to calculate nDNA versus mtDNA distance-differences.

### mtDNA haplotype analysis

For both the 1KGP and the HGDP data, mitochondrial haplotypes were determined from mtDNA sequence variants using the HaploGrep2 program [41]. Evolutionary relationships among mtDNA haplogroups were taken from the PhyloTreemt website [42]. Counts of mtDNA haplogroups were determined for the individual populations, and the counts were hierarchically clustered according to the continental population groups from the 1KGP and HGDP along with the origins of their previously characterized macro-haplogroups. 1KGP mtDNA haplotypes were determined based on 3,892 sequence variants from whole genome sequencing, whereas HGDP mtDNA haplotypes were determined based on 162 variants from SNP arrays.

## Results and discussion

### Comparison of nuclear versus mitochondrial genetic divergence

**Fig. 1:**
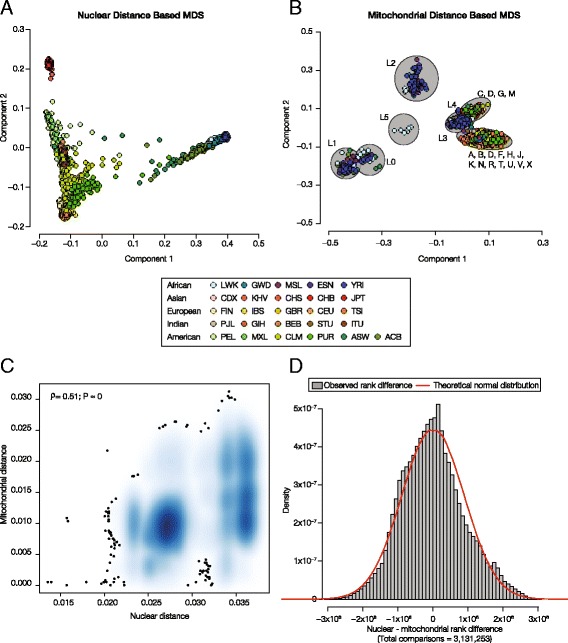
Comparison of nuclear (nDNA) versus mitochondrial (mtDNA) genetic divergence levels. Genetic divergence levels between all pairs of human individuals from the 1KGP were calculated as described in the Materials and Methods. **a** Multidimensional scaling (MDS) plot showing the evolutionary relationships among the 1KGP individuals based on their nuclear (nDNA) genetic distances. **b** MDS plot showing the evolutionary relationships among the 1KGP individuals based on their mitochondrial (mtDNA) genetic distances. Mitochondrial haplogroup designations are shown on the plot and macro-haplogroups are indicated by grey circles. For panels A & B, individuals from different populations are color coded as shown in the key. **c** Density scatterplot showing the regression of nuclear (x-axis) against mitochondrial (y-axis) genetic distances for all pairs of individuals. Denser regions of points are shown in *dark blue*; outlier points are indicated as *black dots*. The Spearman correlation coefficient (*ρ*) and corresponding *P*-value are shown. **d** Distribution of the nuclear versus mitochondrial distance-differences. A theoretical normal distribution (*red line*) is superimposed over the observed distribution (*grey bars*)

The nuclear-mitochondrial mismatch hypothesis rests on the idea that nuclear and mitochondrial genomes co-evolve as populations diverge and thus can be taken to predict that nuclear DNA (nDNA) and mitochondrial DNA (mtDNA) divergence levels will be correlated. In other words, closely related pairs of individuals (from within populations) should show low levels of both nDNA and mtDNA divergence, whereas distantly related individuals (from between populations) should have relatively divergent nuclear and mitochondrial sequences. To evaluate this prediction, we computed the nDNA and mtDNA allele sharing distances between all pairs of individuals from the 1KGP as described in the Materials and Methods. The 1KGP entailed the characterization of nuclear and mitochondrial genome sequences of 2,504 individuals across a broad range of human population genetic diversity: 26 populations representing five major continental population groups (Table 1). Multidimensional scaling (MDS) was used to plot the evolutionary relationships among individuals in two dimensions (components 1 & 2 in Fig. 1a & b) based on the nDNA and mtDNA distances. The genetic distances calculated for nuclear DNA accurately reflect known evolutionary relationships among human populations (Fig. 1a) [29, 30]. African, East Asian and European populations occupy the three poles of human genetic diversity with African populations relatively distinct from the others. Admixed populations from India and the Americas occupy intermediate positions according the relative ancestry contributions from ancient source populations.

Genetic distances calculated from mtDNA sequences also reveal substantial genetic structure among human populations (Fig. 1b). In the case of mtDNA, the major groups correspond very well with previously characterized mtDNA haplogroups (Additional file 1: Figure S1) [42]. The primary MDS component separates the set of ancient L mtDNA haplogroups (L0, L1 & L5) from the more derived L haplogroups (L3 & L4), which cluster with the rest of the derived haplogroups. The MDS clusters of the derived mtDNA haplogroups (M, N & R) also correspond well with the previously known classification; although, the mtDNA genetic distances provided relatively little resolution within these subgroups.

We regressed the nDNA versus mtDNA pairwise genetic distances to test for the correlation predicted by the nuclear-mitochondrial mismatch hypothesis. Overall, nDNA and mtDNA genetic distances are highly correlated, consistent with the prediction (Fig. 1c). Analysis of 2,504 individuals from the 1KGP yields >3 million pairwise comparisons, and the nDNA and mtDNA genetic distances are correlated at *r* = 0.51, *P* ≈ 0. Despite the high overall correlation between nDNA and mtDNA genetic distances, there is a substantial amount of spread in the differences observed for pairs of nDNA versus mtDNA distances (Fig. 1c). There are numerous pairs of individuals, the outliers in the distance-difference distribution (Fig. 1d), that have very closely related nuclear genomes and distantly related mitochondrial genomes or vice versa. These observations point to healthy (viable) individuals that nevertheless have potentially mismatched nuclear and mitochondrial genomes. We attempted to further evaluate this possibility by analyzing the distribution of mtDNA haplotypes among global human populations.

### Global distribution of mtDNA haplotypes

**Fig. 2:**
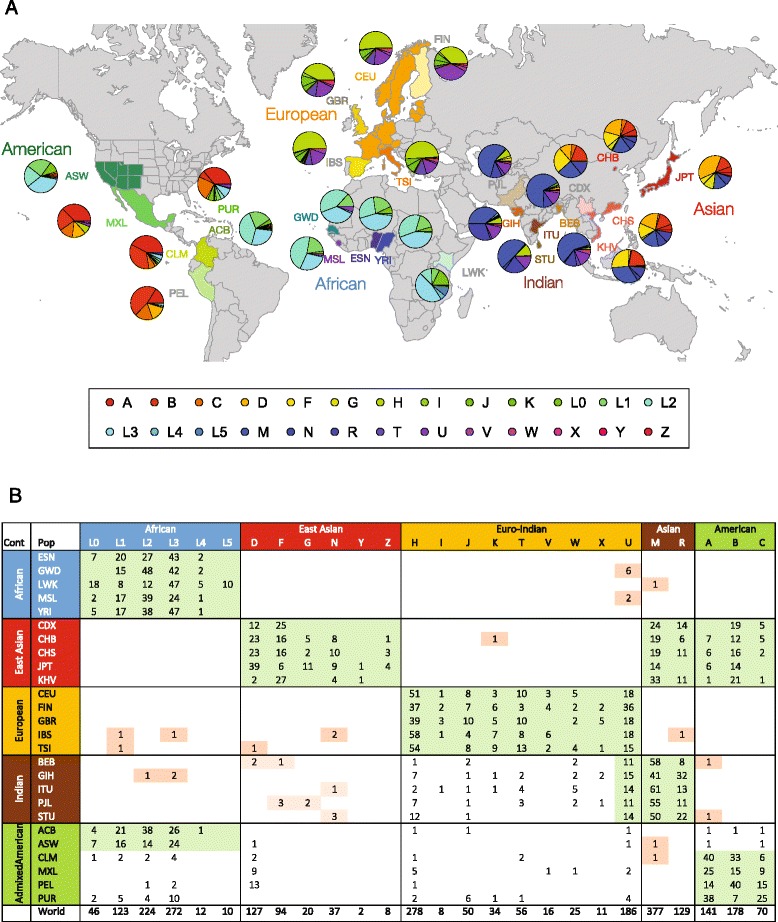
Global distribution of mtDNA haplogroups. **a** Map showing the names and locations of the 1KGP populations studied here along with pie charts showing the relative frequencies of mtDNA haplogroups for each population. The haplogroups are color coded as shown in the key. **b** Counts of mtDNA haplogroups for each 1KGP population. Haplogroup counts are hierarchically clustered along both axes. The y-axis corresponds to the 1KGP continental population groups, and the x-axis corresponds to previously characterized mtDNA macro-haplogroups. The continental origins of the mtDNA macro-haplogroups are shown. Mitochondrial haplogroups that show correspondence (i.e., are matched) between the 1KGP continental population groups and the mtDNA macro-haplogroups are shaded in *green*. Mismatched mtDNA haplogroups are shaded in *orange*

Human mtDNA haplotypes are widely used as markers of maternal ancestry, and accordingly the continental origins of mtDNA haplotype groups are well known [43]. Analysis of nuclear DNA can also be used to resolve evolutionary relationships among human populations and for individual ancestry assignment [29, 30]. The 1KG data (Table 1) provide an opportunity to compare the global distributions of human mitochondrial and nuclear genetic diversity and to test the hypothesis of nuclear-mitochondrial genome incompatibility among naturally occurring populations. Mitochondrial sequence variants for the 1KGP individuals were converted into mtDNA haplotypes using the HaploGrep2 program [41] as described in the Materials and Methods. The distributions of corresponding mtDNA haplogroups were characterized for the 26 populations of the 1KGP as shown in Fig. 2a. The observed global distributions of these mtDNA haplogroups correspond well with the previously characterized origins of mtDNA haplogroups. For example, the ancestral L haplogroup predominates in Africa [44], whereas the D and F haplogroups are most frequent in East Asia [45]. The H, U and T haplogroups are most common in Europe [46].

The observed numbers of each mtDNA haplogroup were recorded for each individual population and clustered into the five major population groups as shown in Fig. 2b. This allowed us to evaluate the extent to which observed mtDNA haplogroups correspond to their expected continent (or broad geographic region) of origin. The African, East Asian and European populations show very coherent patterns of mtDNA haplogroup distributions, whereas the Indian and American population groups show more divergent haplogroups consistent with their admixed origins. Indian populations show a combination of largely European and Asian mtDNA haplogroups, consistent with relatively ancient human migration and admixture events that formed these populations [47, 48]. Interestingly, the Gujarati (GIH) population from Western India shows several instances of African haplogroups, perhaps consistent with subsequent migrations across the Indian Ocean or along the coast. The American populations from the 1KGP were formed by more recent admixture between European, Native American and African source populations [49–51]. Accordingly, individuals from these populations show mtDNA haplogroups corresponding to each of these regions. Native American mtDNA haplogroups are most common among the four admixed Latino populations (CLM, MXL, PEL and PUR), whereas African mtDNA haplogroups are most common among the African-American (ASW) and Afro-Caribbean (ACB) populations. The prevalence of Native American haplotypes in Latino populations is not necessarily correlated with their inferred ancestry based on nuclear DNA. For example, 67% of mtDNA haplotypes from Puerto Rico have a Native American origin, and 13% have a European origin; analysis of nuclear DNA, on the other hand, indicates that the same population has 72% European ancestry compared to only 13% Native American ancestry.

While the co-occurrence of nuclear and mitochondrial genomes with distinct ancestries in admixed populations may be expected, it is nevertheless inconsistent with the nuclear-mitochondrial genome incompatibility hypothesis. Perhaps even more strikingly, there are a number of mismatched nuclear-mitochondrial genome pairs among presumably non-admixed populations. For example, individuals from African populations in Gambia (GWD) and Sierra Leone (MSL) have the U mtDNA haplogroup that is most often found in Europe and India. The specific U mtDNA haplotypes found in these populations all correspond to the U6 haplogroup. This haplogroup has a Near East origin followed by expansion into North Africa [52]. The presence of this haplogroup in West African populations likely reflects subsequent contact with North African groups [53]. A single individual from the Beijing population (CHB) of the East Asian continental group was found to have a K mtDNA haplogroup, which is not known to be found in East Asia [54]. Several individuals from European populations in Spain (IBS) and Italy (TSI) have African L mtDNA haplogroups. This likely reflects relatively ancient African admixture that has been documented for Southern European populations [55]. These same two European populations also have individuals with more typically Asian mtDNA haplotypes from the D, N and R haplogroups. It should be noted that these particular haplogroups are widespread and have previously been found in Europe [45]. This result however underscores the point that members of the same haplogroup can co-occur with nuclear genomes that have very distinct genetic ancestries.

We performed a replication analysis of the global distribution of mtDNA haplotypes using mtDNA sequence variants characterized with SNP arrays as part of the HGDP. While the SNP array data from this project provide substantially less resolution than the sequence data from the 1KGP – 162 mtDNA variants for HGDP compared to 3,892 variants for 1KGP – we were still able to infer mtDNA haplotypes from the HGDP variant data, albeit at a more granular level. Nevertheless, the HGDP data also show a number of cases of nuclear-mitochondrial lineage mismatches, thereby contradicting the nuclear-mitochondrial mismatch hypothesis (Additional file 2: Table S2).

As an additional control analysis, we performed a similar comparison of the distribution of the Y-DNA haplotypes across the populations of the 1KGP. The co-occurrence of Y-DNA and nuclear genomes with distinct ancestries can also be observed for this dataset; however, this appears to occur less often than seen for mtDNA (Additional file 3: Table S3). The slight difference between the mtDNA and Y-DNA results is consistent with previous work showing sex-specific patterns of human migration characterized by relatively lower levels of male migration, and higher levels of female migration, based on the phenomenon of patrilocality [56].

### Phylogenetic discordance of mtDNA haplotypes

**Fig. 3:**
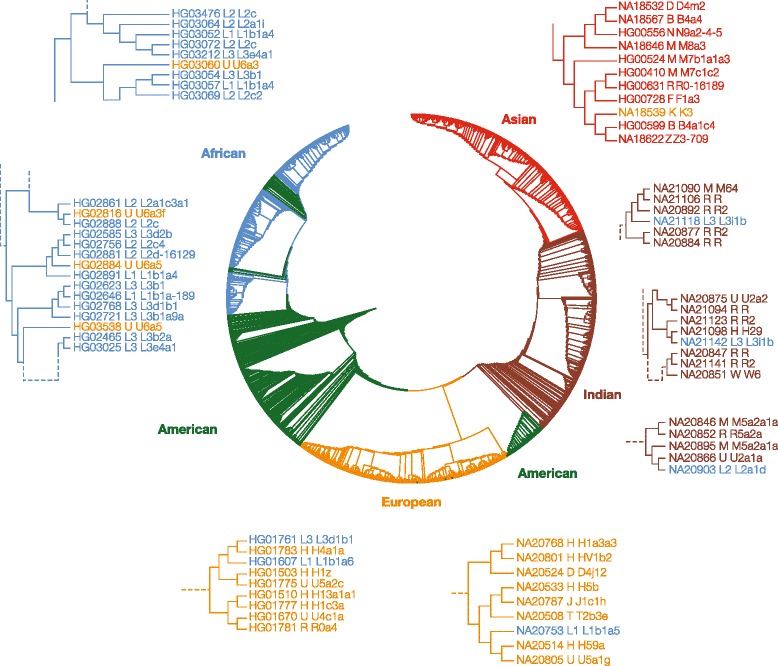
Phylogenetic distribution of mtDNA haplotypes. A phylogeny based on nuclear (nDNA) genetic distances is shown. Branches are color coded, and groups are labeled, according to their 1KGP continental population groups. Individuals’ mtDNA haplotypes are superimposed on the nDNA tree. Subtrees are expanded to show examples of very closely related pairs of individuals (i.e., sister taxa) that have divergent mtDNA haplotypes

Given the existence of a number of population mismatched mtDNA haplotypes, as described in the previous section, we used a phylogenetic approach to more directly compare nDNA genetic distances to the distribution of mtDNA haplotypes. The nDNA genetic distances were used to compute a phylogenetic tree relating all individuals from the 1KGP, and individuals’ mtDNA haplotypes were then considered in the context of this tree (Fig. 3). Populations belonging to the five major continental groups are clearly resolved along this phylogeny, underscoring the extent to which nuclear genetic divergence recapitulates human evolutionary history. The only exception is the placement of the admixed American populations according to their relative ancestry proportions. The African-American populations ASW and ACB group together with the other African populations, whereas the Peruvian population (PEL) occupies an intermediate position owing to its relatively high levels of Native American ancestry. These same patterns can be observed in the nDNA distance MDS plot (Fig. 1a).

The phylogenetic placement of the mtDNA haplotypes also highlights the extent of naturally occurring nuclear-mitochondrial genome mismatch that can be seen for human populations. Pairs of individuals with very low nDNA divergence levels, i.e. sister taxa on the nDNA tree shown in Fig. 3, can have mtDNA haplotypes that are extremely divergent (see mtDNA haplogroup tree in Additional file 1: Figure S1). For example, two European individuals with L1 haplotypes, which correspond to one of the most ancient African mtDNA haplogroups, are most closely related to individuals with the highly derived H mtDNA haplogroup. Similarly, an Indian individual with an ancient African L2 mtDNA haplotype is most closely related to an individual with the highly derived U mtDNA haplotype. In Africa, U mtDNA haplotypes are paired with more ancient L haplotypes reflecting gene flow from the Near East back into Africa as previously discussed.

## Conclusion

The results of our analysis on naturally occurring human genetic variation show that nuclear and mitochondrial genomes from divergent human populations can co-exist within presumably healthy individuals, indicating that such mismatched nDNA-mtDNA combinations are not deleterious and have not been eliminated by purifying selection. In other words, the long and ongoing experiment of human evolution provides no evidence whatsoever in support of the nuclear-mitochondrial mismatch hypothesis. These results can be taken to support the feasibility, and potential safety, of MR-assisted in vitro fertilization, at least with respect to the compatibility of human nDNA and mtDNA genomes. Of course, our results do not bear on any potential complications related to the technical implementation of such a complicated procedure. For example, it is extremely difficult to ensure that none of the defective mitochondria are transferred along with the nuclear genome. Indeed, it was recently shown that even when only a small percentage of defective mitochondria are carried over in the nuclear transfer process, they can increase in copy number and eventually replace most or all of the healthy mitochondria from the egg donor [57]. Such technical hurdles will need to be addressed to ensure the maximum safety of MR therapy.

Our results are in conflict with a number of previous studies on model organisms, which provide numerous lines of evidence in support of nuclear-mitochondrial genome incompatibility. For example, studies in mice have shown physical and neurological deficits related to nuclear-mitochondrial mismatches [14, 15]. In addition, the co-occurrence of divergent nuclear and mitochondrial genomes in invertebrates has been associated with diminished mitochondrial function [25, 26] along with deleterious effects on aging [16–19], survival [20] and fertility [21–24]. When considered together, these previous studies have been taken to issue a strong note of caution against MR therapy [9, 11].

It is interesting to note that much of the resistance to MR therapy has been articulated by evolutionary biologists who emphasize the extent to which nuclear and mitochondrial genomes co-evolve along population lineages [11]. This realization has raised the seemingly legitimate concern that advocates of MR therapy, and/or the regulatory bodies that are charged with evaluating its safety, may not have adequately considered the implications of evolution for the implementation of this new technology. However, the results of our study suggest that the model organism studies that have been used to argue against the safety of MR therapy do not accurately reflect the nature of human evolution [27]. For the most part, these model organism studies relied on backcrossing and the generation of inbred lines, and they also involved relatively divergent populations. Experiments of this kind can be expected to result in extremes of nuclear-mitochondrial genome divergence. Human populations, on the other hand, tend to show both low levels of genetic divergence and low inbreeding. Accordingly, one may expect to see less pronounced effects of nuclear-mitochondrial mismatch in human populations, and that is exactly what we observed in our study.

## Additional files

Additional file 1: Table S1. HGDP populations analyzed in this study. **Figure S1**. (A) Phylogenetic tree based on mtDNA haplotype genetic distances and (B) dendogram showing previously defined relationships among major mtDNA haplogroups. (DOCX 344 kb)
Additional file 2: Table S2. Counts of mtDNA haplogroups for the HGDP populations analyzed here. Global population distributions of mtDNA haplogroups are organized as shown for the 1KGP in Table 1. (XLSX 14 kb)
Additional file 3: Table S3. Counts of Y-DNA haplogroups for the 1KGP populations analyzed here. Global population distributions of Y-DNA haplogroups are organized as shown for the mtDNA haplogroups in Table 1. (XLSX 13 kb)

## Abbreviations

1KGP: 1000 genomes project
HFEA: Human fertilisation and embryology authority
IVF: in vitro fertilization
MDS: Multidimensional scaling
MR: Mitochondrial replacement
mtDNA: mitochondrial DNA
nDNA: nucleosomal DNA
