# Supplementary material for: Implications of human evolution and admixture for mitochondrial replacement therapy
Source: BMC Genomics. 2017 Feb 8;18:140. doi: 10.1186/s12864-017-3539-3 (PMC5299762; doi:10.1186/s12864-017-3539-3)

Additional file for:

**Implications of human evolution and admixture for mitochondrial replacement therapy**

*Lavanya Rishishwar and I. King Jordan*

Table S1. **Individuals analyzed in this study from the HGDP-CEPH**. The continental groups, country, population name and number of individuals from each population are shown.

|  | Country | Population | N |  | Country | Population | N |
| --- | --- | --- | --- | --- | --- | --- | --- |
| **Africa (n=127)** | Kenya | Bantu N.E. | 12 | **East Asia (n=241)** | Cambodia | Cambodians | 11 |
|  | South Africa | Bantu S.E. Pedi | 1 |  | China | Dai | 10 |
|  | South Africa | Bantu S.E. S.Sotho | 1 |  | China | Daur | 10 |
|  | South Africa | Bantu S.E. Tswana | 2 |  | China | Han | 35 |
|  | South Africa | Bantu S.E. Zulu | 1 |  | China | Han N | 10 |
|  | South Africa | Bantu S.W. Herero | 2 |  | China | Hezhen | 10 |
|  | South Africa | Bantu S.W. Ovambo | 1 |  | Japan | Japanese | 31 |
|  | C.A.R. | Biaka Pygmies | 36 |  | China | Lahu | 10 |
|  | Senegal | Mandenka | 24 |  | China | Miaozu | 10 |
|  | D.R.C. | Mbuti Pygmies | 15 |  | China | Mongola | 10 |
|  | Namibia | San | 7 |  | China | Naxi | 10 |
|  | Nigeria | Yoruba | 25 |  | China | Oroqen | 10 |
| **America (n=108)** | Colombia | Colombians | 13 |  | China | She | 10 |
|  | Brazil | Karitiana | 24 |  | China | Tu | 10 |
|  | Mexico | Maya | 25 |  | China | Tujia | 10 |
|  | Mexico | Pima | 25 |  | China | Xibo | 9 |
|  | Brazil | Surui | 21 |  | Siberia | Yakut | 25 |
| **Central South Asia (n=210)** | Pakistan | Balochi | 25 |  | China | Yizu | 10 |
|  | Pakistan | Brahui | 25 | **Europe (n=161)** | Russia Caucasus | Adygei | 17 |
|  | Pakistan | Burusho | 25 |  | France | French | 29 |
|  | Pakistan | Hazara | 25 |  | France | French Basque | 24 |
|  | Pakistan | Kalash | 25 |  | Italy (Bergamo) | North Italian | 14 |
|  | Pakistan | Makrani | 25 |  | Orkney Islands | Orcadian | 16 |
|  | Pakistan | Pathan | 25 |  | Russia | Russian | 25 |
|  | Pakistan | Sindhi | 25 |  | Italy | Sardinian | 28 |
|  | China | Uygur | 10 |  | Italy | Tuscan | 8 |
|  |  |  |  | **Middle East (n=178)** | Israel (Negev) | Bedouin | 49 |
|  |  |  |  |  | Israel (Carmel) | Druze | 48 |
|  |  |  |  |  | Algeria (Mzab) | Mozabite | 30 |
|  |  |  |  |  | Israel (Central) | Palestinian | 51 |
|  |  |  |  | **Oceania (n=39)** | Bougainville | NAN Melanesian | 22 |
|  |  |  |  |  | New Guinea | Papuan | 17 |

Figure S1. **Phylogenetic tree based on the mtDNA distances.** (A) Neighbor-joining phylogeny of mtDNA haplotypes reconstructed based on their mtDNA allele sharing sequence distances. Haplotype designations are shown for the leaves of the tree (the tips of each terminal branch), and the locations of the major mtDNA haplogroups (L3, M, N & R) are indicated on internal branches. A scale bar corresponding to the allele sharing distance is shown. (B) A dendrogram showing the previously defined relationships among mtDNA haplotypes and the major mtDNA haplogroups [40].


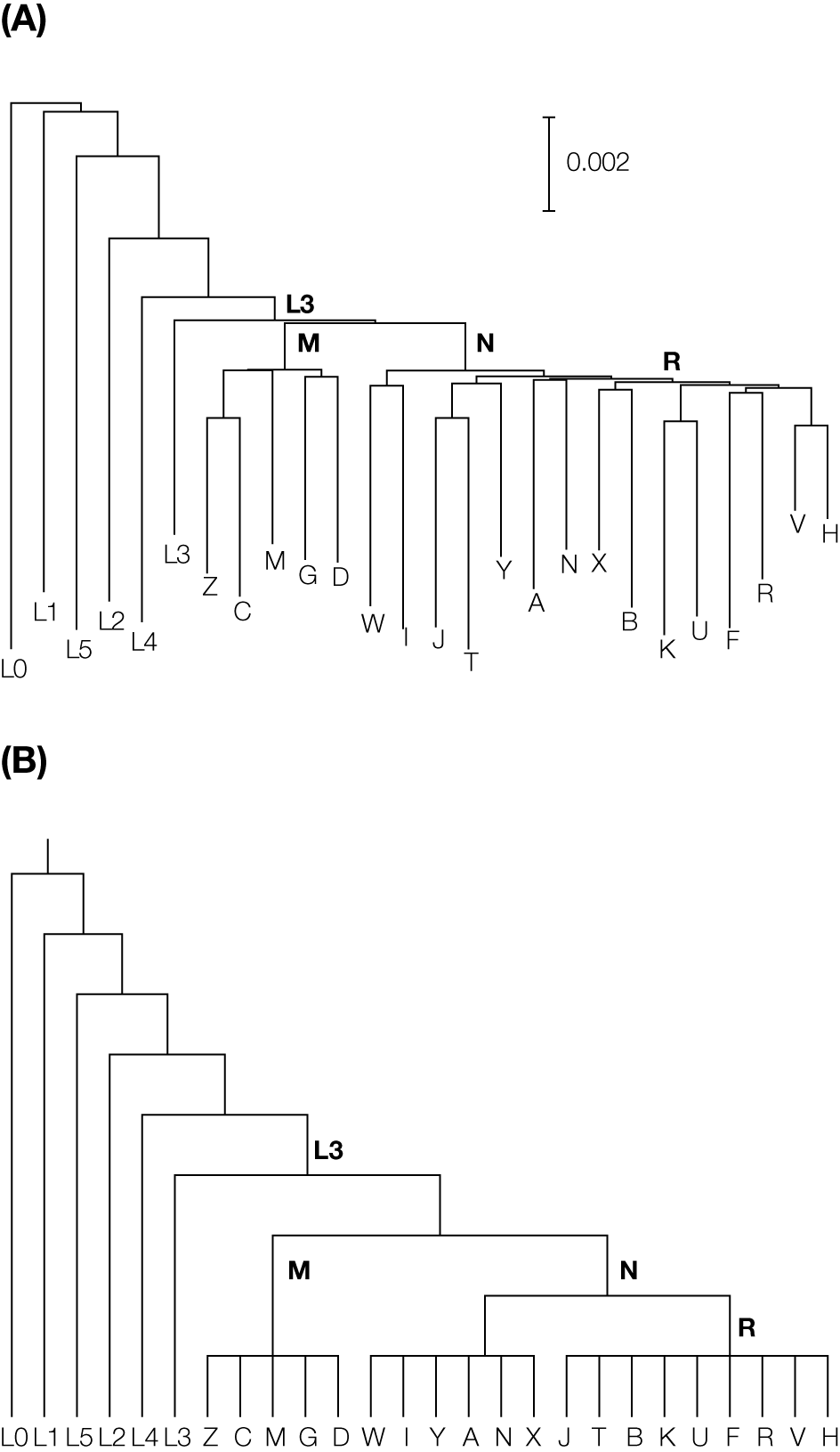

Supplement: Additional file 1: Table S1. — HGDP populations analyzed in this study. Figure S1. (A) Phylogenetic tree based on mtDNA haplotype genetic distances and (B) dendogram showing previously defined relationships among major mtDNA haplogroups. (DOCX 344 kb) [file 12864_2017_3539_MOESM1_ESM.docx]
